# Glucocorticoid receptor antagonism reverts docetaxel resistance in human prostate cancer

**DOI:** 10.1530/ERC-15-0343

**Published:** 2016-01

**Authors:** Jan Kroon, Martin Puhr, Jeroen T Buijs, Geertje van der Horst, Daniëlle M Hemmer, Koen A Marijt, Ming S Hwang, Motasim Masood, Stefan Grimm, Gert Storm, Josbert M Metselaar, Onno C Meijer, Zoran Culig, Gabri van der Pluijm

**Affiliations:** 1 Department of Urology, Leiden University Medical Center, Albinusdreef 22333 ZA, Leiden, The Netherlands; 2 Department of Targeted Therapeutics, MIRA Institute for Biological Technology and Technical Medicine, University of Twente, Enschede, The Netherlands; 3 Department of Urology, Medical University of Innsbruck, Innsbruck, Austria; 4 Department of Clinical Oncology, Leiden University Medical Center, Leiden, The Netherlands; 5 Division of Experimental Medicine, Imperial College London, London, UK; 6 Department of Pharmaceutics, Utrecht Institute for Pharmaceutical Sciences, Utrecht University, Utrecht, The Netherlands; 7 Department of Endocrinology, Leiden University Medical Center, Leiden, The Netherlands

**Keywords:** docetaxel, glucocorticoid receptor, prostate cancer, therapy resistance

## Abstract

Resistance to docetaxel is a major clinical problem in advanced prostate cancer (PCa). Although glucocorticoids (GCs) are frequently used in combination with docetaxel, it is unclear to what extent GCs and their receptor, the glucocorticoid receptor (GR), contribute to the chemotherapy resistance. In this study, we aim to elucidate the role of the GR in docetaxel-resistant PCa in order to improve the current PCa therapies. GR expression was analyzed in a tissue microarray of primary PCa specimens from chemonaive and docetaxel-treated patients, and in cultured PCa cell lines with an acquired docetaxel resistance (PC3-DR, DU145-DR, and 22Rv1-DR). We found a robust overexpression of the GR in primary PCa from docetaxel-treated patients and enhanced GR levels in cultured docetaxel-resistant human PCa cells, indicating a key role of the GR in docetaxel resistance. The capability of the GR antagonists (RU-486 and cyproterone acetate) to revert docetaxel resistance was investigated and revealed significant resensitization of docetaxel-resistant PCa cells for docetaxel treatment in a dose- and time-dependent manner, in which a complete restoration of docetaxel sensitivity was achieved in both androgen receptor (AR)-negative and AR-positive cell lines. Mechanistically, we demonstrated down-regulation of Bcl-xL and Bcl-2 upon GR antagonism, thereby defining potential treatment targets. In conclusion, we describe the involvement of the GR in the acquisition of docetaxel resistance in human PCa. Therapeutic targeting of the GR effectively resensitizes docetaxel-resistant PCa cells. These findings warrant further investigation of the clinical utility of the GR antagonists in the management of patients with advanced and docetaxel-resistant PCa.

## Introduction

Resistance to chemotherapy is a major hurdle in prostate cancer (PCa) treatment. Although chemotherapeutic treatments typically display initial benefit, cancer cells frequently acquire novel characteristics that will render these cells unresponsive to current cytotoxic treatments. Docetaxel (Taxotere, Sanofi-Aventis, Paris, France) is a microtubule-stabilizing agent that is clinically approved for a range of malignancies, including castration-resistant PCa (CRPC) for which it is the standard-of-care and prolongs survival of patients (Tannock *et al*. 2004). Unfortunately, tumors inevitably progress due to the acquired docetaxel resistance (O'Neill *et al*. 2011). In addition, docetaxel resistance is often accompanied with a cross-resistance, i.e., dampened efficacy of other antitumor therapeutics and conversely, the use of other therapeutic agents, e.g., antiandrogens, appears to be associated with the emergence of resistance to docetaxel (van Soest *et al*. 2014). Therefore, identification of the underlying molecular mechanisms of docetaxel resistance is of a pivotal importance to combat docetaxel resistance in clinics (Madan *et al*. 2011).

The glucocorticoid receptor (GR) is a receptor that, upon binding of glucocorticoids (GCs) (e.g. dexamethasone (DEX)), regulates gene expression. GC binding to the GR leads to a rapid GR dimerization and subsequent nuclear translocation where it interacts with the GC response elements or transcription factors (Lewis-Tuffin & Cidlowski 2006). Although GCs are frequently administered to patients with advanced PCa, mainly for antiemetic purposes, their usage remains controversial as both pro- and antitumor effects have been described (Montgomery *et al*. 2014). On the one hand, direct antitumor efficacy of DEX has been described in preclinical (Kroon *et al*. 2015) and clinical studies (Venkitaraman *et al*. 2008). On the other hand, GC usage seems to be associated with the resistance to antiandrogen therapy (Arora *et al*. 2013, Isikbay *et al*. 2014) and chemotherapy (Zhang *et al*. 2007). Simultaneous DEX administration was shown to undermine the antitumor effects of paclitaxel *in vitro* and *in vivo*, thus giving rise to chemotherapy resistance (Zhang *et al*. 2007). Our hypothesis holds that GR antagonism may lead to the reversal of chemotherapy resistance. In this study we describe that elevated GR expression can mediate chemotherapy resistance, and that interference with the canonical GR signaling counteracts docetaxel resistance in PCa, thus resensitizing PCa cells for this chemotherapeutic agent.

## Materials and methods

### Tissue microarray and immunohistochemistry

To examine GR expression in docetaxel-treated PCa tissue specimens, we used a tissue microarray (TMA) of formalin-fixed, paraffin-embedded tissue blocks of 14 PCa patients who underwent neoadjuvant chemotherapy with docetaxel before radical prostatectomy and of 14 untreated PCa patients (Puhr *et al*. 2012, 2014). Every patient is represented with three cancer and three benign cores on the TMA. The use of archived material was approved by the Ethics Committee of the Medical University of Innsbruck (study no. AM 3174 including amendment 2). Patients received no other chemotherapeutics or antiandrogens prior to the radical prostatectomy, and both groups were matched for Gleason score and age. Patient characteristics, TMA assembling, and staining protocols were performed as previously described (Puhr *et al*. 2012). The TMA was stained with anti-GR (1:200, D6H2L, Cell Signaling Technology Inc, Danvers, MA, USA).

Immunohistochemistry was performed with a Discovery – XT staining device (Ventana, Tucson, AZ, USA) and images were captured with a Zeiss Imager Z2 microscope (Zeiss, Vienna, Austria) equipped with Pixelink PL-B622-CU camera (Canimpex Enterprises Ltd, Halifax, NS, Canada). The scoring of the GR expression was performed as follows: the cores were scored for GR intensity (no signal (0), weak signal (1), moderate signal (2), and strong signal (3)) and the percentage of positive cells (0% (0), 0–25% (1), 25–50% (2), 50–75% (3), and over 75% (4)). The immune reactivity score was calculated with the following formula: intensity score * positive cell score and the average of three cores per patient is depicted. Statistical differences between both groups were calculated with the Mann–Whitney *U* test.

### Cell culture and reagents

PC3, DU145, and 22Rv1 cells were cultured in RPMI-1640 supplemented with FCS, penicillin/streptomycin, and glutamine. Docetaxel-resistant cells (PC3-DR, DU145-DR, and 22Rv1-DR) were generated by increasing exposure to docetaxel and subsequently cultured under the presence of 12.5 nM docetaxel (O'Neill *et al*. 2011, Puhr *et al*. 2012). The identity of cell lines was confirmed by short tandem repeat analysis and cell line passages used in all experiments ranged from p2 to p17. Docetaxel, RU-486 (mifepristone), verapamil (all Sigma–Aldrich), and ABT-263 (Selleckchem, Huissen, The Netherlands) were dissolved in EtOH, DEX (Buma, Uitgeest, The Netherlands) in PBS and cyproterone acetate (CPA, Sigma–Aldrich) in MeOH.

### CRISPR/CAS9

sgRNA sequences (sgRNA_GR1: ACGGCTGGTCGACCTATTG) were designed using the CRISPR Design Tool (http://crispr.mit.edu) to specifically target exon 2 of the GR-1 gene and were cloned into a PCR expression vector (Addgene: 41824). PC3-DR were transfected with CAS9 WT (Addgene: 41815) and sgRNA-GR1 plasmid (2 μg/plasmid) using Fugene (Promega). The sensitivity to docetaxel was assessed in transiently transfected cells and in stable cell lines, generated by expansion of single cells. Knockdown efficiency was determined using western blot.

### Real-time qPCR

Cells were incubated with DEX in combination with RU-486 or CPA for 6 h and mRNA was isolated using Tripure (Invitrogen). cDNA was synthesized by RT (Promega). Real-time qPCR was performed on the Bio-Rad IQ5 Cycler and gene expression was normalized to the expression of GAPDH. The primer sequences are: GAPDH, F: 5′-GACAGTCAGCCGCATCTTC-3′; GAPDH, R: 5′-GCAACAATATCCACTTTACCAGAG-3′; GILZ. F: 5′-GCACAATTTCTCCATCTCCTTCTT-3′; GILZ, R: 5′-TCAGATGATTCTTCACCAGATCCA-3′; FKBP5, F: 5′-GAATGGTGAGGAAACGCCGAT-3′; FKBP5, R: 5′-TGCCAAGACTAAAGACAAATGGT-3′; and P-glycoprotein (P-gp), F: 5′-CCCATCATTGCAATAGCAGG-3′; P-gp, R: 5′-GTTAAACTTCTGCTCCTGA-3′.

### Western blot

Cells were lysed in RIPA buffer and protein was loaded on a SDS–PAGE gel and subsequently transferred to a nitrocellulose membrane. Membranes were incubated with the primary antibody overnight at 4 °C and with the secondary antibody for 1 h at room temperature. The following antibodies were used: anti-GR (1:500, sc-8992, Santa Cruz Biotechnology, Santa Cruz, CA, USA), anti-GAPDH (1:10 000, Chemicon, Temecula, CA, USA), anti-β-actin (1:5000, Sigma), anti-Bcl-xL (1:1000, sc-23958, Santa Cruz Biotechnology), and anti-Bcl-2 (1:1000, sc-7382, Santa Cruz Biotechnology).

### Viability assay and clonogenic assay

For the viability assay, cells were seeded 1500 cells/well and were exposed to docetaxel in combination with RU-486 and/or CPA. Cell viability was measured using the MTS assay (Celltiter 96 Aqueous One Solution Cell Proliferation Assay; Promega). For the clonogenic assay, cells were seeded 100 cells/well and exposed to a combination of docetaxel and RU-486. After 10–14 days, wells were fixed with 4% paraformaldehyde, and colonies were stained using crystal violet.

### Cell death analysis

Cell death was assessed using an Annexin V/propidium iodine (PI) assay (Invitrogen). PC3-DR or DU145-DR cells were seeded 200 000/well and treated with a combination of 30 nM docetaxel and 3 μM RU-486. Floating and adherent cells were harvested and incubated with FITC–Annexin V and PI before analysis with flow cytometry. Viable cells were defined as Annexin V^−^/PI^−^, early apoptotic cells as Annexin V^+^/PI^−^ and late apoptotic/necrotic cells as Annexin V^+^/PI^+^.

### Side population

The drug efflux properties of cells were determined as previously described (Golebiewska *et al*. 2011). In short, cells were incubated with 3 μM RU-486, 10 μM CPA, or 0.05–5.0 μM P-gp inhibitor verapamil prior to addition of Hoechst 33342 (5 μg/ml, Sigma–Aldrich). To-Pro-3 (0.5 μM, Life Technologies) was added to exclude dead cells. Hoechst 33342 exclusion was measured with the LSRII using 450 nm (Hoechst blue) and 675 nm (Hoechst red) filters after excitation with a 350 nm u.v. light.

### Statistical analysis

All experimental data are presented as the mean+s.e.m. and represent three independent experiments. Significance was calculated using GraphPad Prism 5.0 Software (San Diego, CA, USA) using either *t*-test or two-way ANOVA with Bonferroni's post testing.

## Results

### GR expression is enhanced in prostate tumors of docetaxel-treated patients and functionally involved in docetaxel resistance *in vitro*

**Figure 1:**
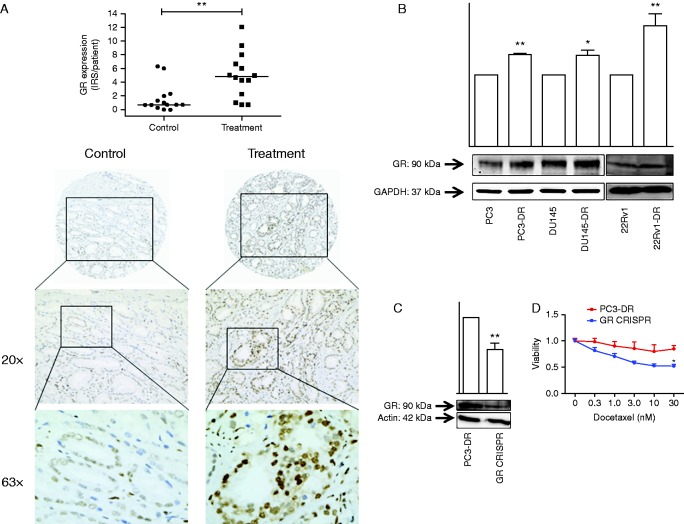
Glucocorticoid receptor (GR) expression is enhanced in docetaxel-treated patients and docetaxel-resistant cell lines and is functionally involved in chemotherapy resistance. (A) Tissue microarray (TMA) analysis revealed the GR overexpression in prostate cancer (PCa) patient tissues treated with neoadjuvant docetaxel compared with chemonaive patients. **P<0.01. (B) Protein expression analysis revealed that GR is overexpressed in docetaxel-resistant cell lines PC3-DR, DU145-DR, and 22Rv1-DR compared with their parental counterparts. *P<0.05 vs parental and **P<0.01 vs parental. (C) Transient CRISPR/CAS9-directed deletion of the GR-1 results in reduced GR expression. **P<0.01 vs PC3-DR. (D) Enhanced docetaxel sensitivity in PC3-DR GR CRISPR cells. *P<0.05 vs PC3-DR.

In this study, we aim to elucidate the role of the GR in docetaxel resistance. To this end, a TMA of 14 docetaxel-treated patients and 14 chemonaive patients was evaluated. Immunohistochemical analysis revealed a significant upregulation of the GR in PCa cells of docetaxel-treated patients compared with the chemonaive patients (*P*<0.01; Fig. 1A). In addition, docetaxel-resistant cell lines PC3-DR, DU145-DR, and 22Rv1-DR, largely unresponsive to docetaxel concentrations of up to 30 nM (independent of serum GC; Supplementary Figure 1, see section on supplementary data given at the end of this article), displayed elevated expression of the GR protein levels when compared with their chemonaive counterparts (Fig. 1B). To evaluate the functional involvement of the GR in docetaxel resistance, we employed the CRISPR/CAS9-mediated knockout technology. Transient transfection of PC3-DR cells with GR-1-targeted sgRNA resulted in reduced GR expression (Fig. 1C) and, strikingly, enhanced sensitivity to docetaxel (−39% viability in PC3-DR CRISPR GR compared with PC3-DR wt upon treatment with 30 nM docetaxel, *P*<0.05; Fig. 1D). In addition, in PC3-DR cells with stable GR knockout (Supplementary Figure 2A), that exhibit similar basal growth rates (Supplementary Figure 2B), docetaxel displays enhanced antitumor efficacy compared with PC3-DR wt cells (Supplementary Figure 2C).

### Therapeutic targeting of the GR completely antagonizes DEX-induced transcriptional activity and strongly resensitizes docetaxel-resistant cells to docetaxel treatment

**Figure 2:**
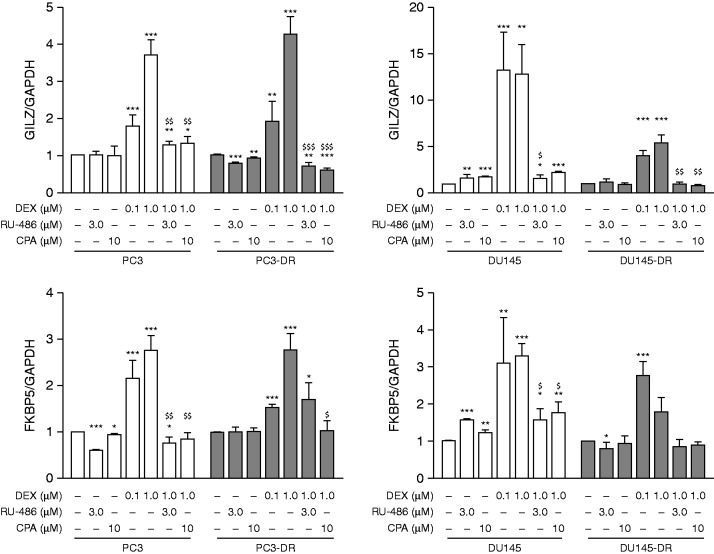
Dexamethasone (DEX)-induced transcriptional activity of the GR-target genes GILZ and FKBP5 is inhibited by RU-486 and CPA. Expression of the GR-target genes GILZ and FKBP5 in PC3-(DR) and DU145-(DR) cells upon treatment with DEX and/or RU-486 or CPA. *P<0.05 vs vehicle; **P<0.01 vs vehicle; ***P<0.001 vs vehicle; ^$^ *P*<0.05 vs 1.0 μM DEX; ^$$^ *P*<0.01 vs 1.0 μM DEX; and ^$$$^ *P*<0.01 vs 1.0 μM DEX.

**Figure 3:**
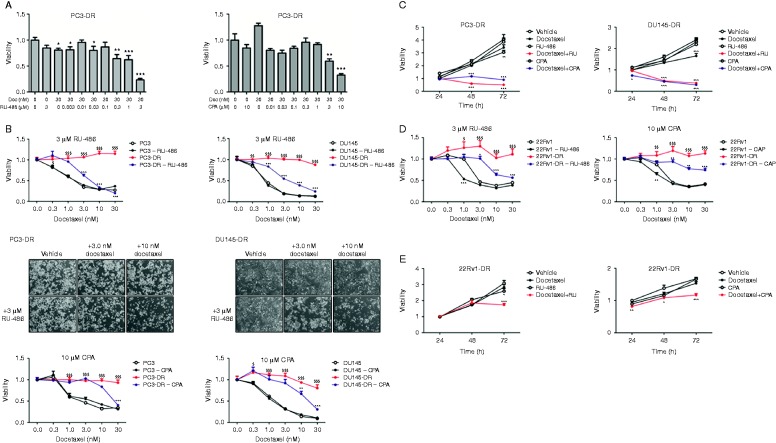
Therapeutic targeting of the glucocorticoid receptor (GR) resensitizes docetaxel-resistant cell lines to docetaxel. (A) GR antagonism with RU-486 or CPA resensitizes PC3-DR cells for docetaxel treatment after 72 h of treatment. *P<0.05 vs vehicle; **P<0.01 vs vehicle; and ***P<0.001 vs vehicle. (B) Dose-dependent (72 h) and (C) time-dependent (30 nM) antitumor effect of docetaxel upon simultaneous treatment with 3 μM RU-486/10 μM CPA and docetaxel in PC3-(DR) and DU145-(DR) cells. ^$^ *P*<0.05 vs parental; ^$$^ *P*<0.01 vs parental; ^$$$^ *P*<0.001 vs parental; **P<0.01 vs docetaxel-resistant line; and ***P<0.001 vs docetaxel-resistant line. (D) GR antagonism with RU-486 or CPA resensitizes AR-positive cell line 22Rv1-DR for docetaxel treatment after 72 h of treatment. ^$^ *P*<0.05 vs parental; ^$$^ *P*<0.01 vs parental; ^$$$^ *P*<0.001 vs parental; *P<0.05 vs docetaxel-resistant line; **P<0.01 vs docetaxel-resistant line; and ***P<0.001 vs docetaxel-resistant line. (E) Time-dependent (30 nM) antitumor effect of docetaxel upon simultaneous treatment with 3 μM RU-486 or 10 μM CPA. *P<0.05 vs vehicle; **P<0.01 vs vehicle; and ***P<0.001 vs vehicle. A full colour version of this figure is available at http://dx.doi.org/10.1530/ERC-15-0343.

The induction of the GR signaling upon exposure with GR agonist DEX was examined by expression analysis of the GR-target genes GR-induced leucine zipper (GILZ) and FK506 binding protein 5 (FKBP5). DEX effectively induced GILZ and FKBP5 expression which was fully reversed by co-incubation with RU-486 (3 μM) and CPA (10 μM) (Fig. 2), indicating of a complete blockage of the GR-activity by the GR-antagonists. To examine if modulation of the GR-activity affects sensitivity to docetaxel, docetaxel-resistant cell lines were incubated with GR-antagonists RU-486 or CPA. Exposure to RU-486 or CPA alone did not influence cell viability of parental (PC3 and DU145) and docetaxel-resistant cell lines (PC3-DR and DU145-DR) (Supplementary Figure 3A, see section on supplementary data given at the end of this article). Strikingly, incubation with both GR-antagonists strongly sensitized docetaxel-resistant cells to docetaxel treatment at doses 0.3–3 μM for RU-486 and 3–10 μM for CPA (Fig. 3A). For further experiments, the doses 3 μM RU-486 and 10 μM CPA were chosen as these showed a strong reduction in cell viability of PC3-DR cells (80 and 70% respectively) upon treatment with 30 nM docetaxel (*P*<0.001) (Fig. 3A). Antagonizing GR-mediated signaling activity significantly resensitized docetaxel-resistant cells to docetaxel treatment dose-dependently (Fig. 3B) and in a time-dependent fashion (*P*<0.001 at 48–72 h) (Fig. 3C) while no effect was observed in docetaxel-sensitive tumor cells (Fig. 3B). In concordance with these findings, combined treatment with RU-486 and docetaxel largely diminished the clonogenic potential of both docetaxel-resistant cell lines (Supplementary Figure 3C). As PCa is fundamentally androgen receptor (AR) driven, we performed similar studies in AR-positive docetaxel-resistant cells. To this end, we utilized the 22Rv1 lineage, which expresses both full length AR as well as splice variant AR-V7 (Shiota *et al*. 2014). In concordance with our results in AR-negative cell lines, 22Rv1-DR cells are also resensitized to docetaxel upon RU-486 and CPA treatment in a dose-dependent (Fig. 3D) and time-dependent manner (Fig. 3E). These observations support a general role of the GR in the development of docetaxel resistance independently of AR status.

### GR antagonism downregulates antiapoptotic Bcl-2 and Bcl-xL proteins

**Figure 4:**
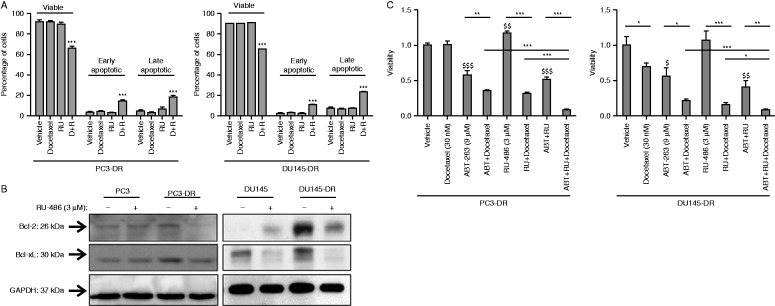
Glucocorticoid receptor (GR) antagonism downregulates the expression of antiapoptotic Bcl-2 and Bcl-xL proteins. (A) Docetaxel-resistant cells undergo apoptosis upon treatment with RU-486 (3 μM) and docetaxel (30 nM). ***P<0.001 vs vehicle. (B) Bcl-2 and Bcl-xL are upregulated in docetaxel-resistant cell lines. Treatment with RU-486 (3 μM) reverses the elevated expression of Bcl-2 and Bcl-xL in docetaxel-resistant prostate cancer cells. (C) Co-incubation with an antagonist for Bcl-2 and Bcl-xL, ABT-263 (9 μM), sensitizes PC3-DR and DU145-DR cells to docetaxel treatment. *P<0.05; **P<0.01; ***P<0.001; ^$^ *P*<0.05 vs vehicle; ^$$^ *P*<0.01 vs vehicle; and ^$$$^ *P*<0.001 vs vehicle.

To further elucidate the mechanism of cell death observed above, we performed an Annexin V/PI analysis. Treatment of docetaxel-resistant cells with 3 μM RU-486 and 30 nM docetaxel resulted in a decrease of viable cells and an increase in early and late apoptotic cells (*P*<0.001; Fig. 4A). Western blot analysis of antiapoptotic proteins revealed an upregulation of Bcl-2 and Bcl-xL, well-known for their inhibitory role in Bak/Bax-mediated cytochrome *c* release in the intrinsic apoptotic pathway, in docetaxel-resistant cell lines compared with their chemonaive counterparts (Fig. 4B). Interestingly, GR antagonism resulted in decreased expression of antiapoptotic Bcl-xL and Bcl-2 in both docetaxel-resistant cells (Fig. 4B). This suggests that the sensitizing effects of the GR antagonism may be partially mediated via modulation of the Bcl-2/Bcl-xL axis. To further explore this, a selective antagonist for Bcl-2 and Bcl-xL was investigated: ABT-263. Treatment with ABT-263 already induced cell death in PC3-DR and DU145-DR cell lines (Fig. 4C). On top of this, ABT-263 significantly resensitized both docetaxel-resistant cell lines to docetaxel treatment (Fig. 4C). Since this effect with ABT-263 was not as potent as the effect observed with RU-486, other mechanisms in addition to Bcl-xL/Bcl-2 downregulation are presumably involved in the resensitization upon the GR inhibition. This notion is supported by the observation that the sensitivity to docetaxel is enhanced in both docetaxel-resistant cell lines if treated with both RU-486 and ABT-263 compared to RU-486 or ABT-263 alone (Fig. 4C).

### Chemotherapy resensitization with RU-486 and CPA cannot be attributed solely to inhibition of P-gp activity

**Figure 5:**
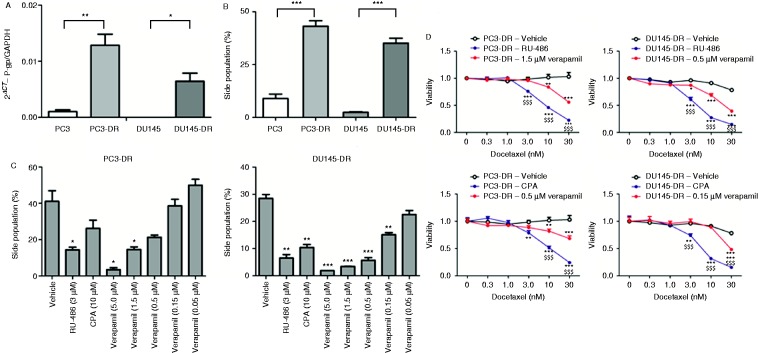
RU-486 and CPA outperform verapamil at dosages with similar P-glycoprotein (P-gp) inhibitory action. (A) P-gp mRNA expression and (B) basal activity in chemo-resistant and sensitive prostate cancer cells. *P<0.05; **P<0.01; and ***P<0.001. (C) Head-to-head comparison of the GR antagonists and verapamil on P-gp activity. *P<0.05 vs vehicle; **P<0.01 vs vehicle; and ***P<0.001 vs vehicle. (D) Head-to-head comparison of the GR antagonists and verapamil on cell viability. *P<0.05 vs vehicle; **P<0.01 vs vehicle; ***P<0.001 vs vehicle; and ^$$$^ *P*<0.001 vs verapamil. A full colour version of this figure is available at http://dx.doi.org/10.1530/ERC-15-0343.

Previously, an inhibitory effect of RU-486 (Gruol *et al*. 1994) and CPA (Frohlich *et al*. 2004) on P-gp activity was described, which could potentially underlie the observed resensitization with the GR antagonists as both PC3-DR and DU145-DR display enhanced P-gp expression (Fig. 5A). To address this, we examined the so called side population, a population of stem-like tumor cells with high P-gp activity leading to the exclusion of P-gp substrate Hoechst. This revealed an enhanced P-gp activity in both docetaxel-resistant cell lines compared to their parental counterparts (Fig. 5B). As expected, RU-486 and CPA display an inhibitory effect on P-gp activity (Fig. 5C). To dissect the resensitizing activities of RU-486 and CPA, we compared their resensitizing capacity to verapamil, a known P-gp inhibitor. Verapamil dosages with equal P-gp inhibitory activity as RU-486 (1.5 μM in PC3-DR and 0.5 μM in DU145-DR) and CPA (0.5 μM in PC3-DR; 0.15 μM in DU145-DR) (Fig. 5C) were compared head-to-head in a viability assay. This revealed a significantly stronger sensitization capacity of both RU-486 and CPA compared to the corresponding verapamil dosages (Fig. 5D), suggesting that a significant proportion of sensitization is indeed mediated via direct inhibition of the GR rather than via P-gp.

## Discussion

In this study, we describe a key role of the GR in PCa docetaxel resistance. We show that the GR is overexpressed in clinical PCa specimens treated with neoadjuvant docetaxel, as well as in docetaxel-resistant PCa cell lines *in* *vitro*. While untreated PCa specimens only display the modest GR levels (Yemelyanov *et al*. 2007, Szmulewitz *et al*. 2012), androgen ablation therapy or chemotherapy result in upregulation and nuclear localization of the GR (Szmulewitz *et al*. 2012, Yemelyanov *et al*. 2012), suggesting the functional involvement of the GR in PCa therapy resistance. Our data fully support this notion and prompted us to further investigate the GR as a therapeutic target in docetaxel-resistant PCa.

Our study reveals a strong resensitizing effect to docetaxel in both AR-negative and AR-positive cell lines upon treatment with the GR antagonists (at dosages that fully antagonize DEX-induced expression of the GR-target genes), suggesting functional involvement of the GR in mediating clinical docetaxel resistance in human PCa. This is supported by recent studies describing the ability of the GR antagonist RU-486 to potentiate the antitumor efficacy of chemotherapeutics in triple-negative breast cancer (paclitaxel; Skor *et al*. 2013) and cervical cancer (cisplatin; Jurado *et al*. 2009). Conversely, stimulation of the GR activity by GR exposure (Herr *et al*. 2003) or stress induction (Reeder *et al*. 2015) confers resistance to chemotherapeutics, supporting the notion of the GR involvement in chemotherapy resistance.

A range of mechanisms underlying docetaxel resistance have been described including: upregulation of antiapoptotic proteins (Yoshino *et al*. 2006), overexpression of ABC transporters (Sanchez *et al*. 2009), aberrant activation of NF-κB (O'Neill *et al*. 2011), alterations in β-tubulin isotypes (Galletti *et al*. 2007), and expression of AR-V7 (Thadani-Mulero *et al*. 2014). GCs were shown to contribute to chemotherapy resistance via upregulation of antiapoptotic proteins (Herr *et al*. 2007). As such, GR-target gene GILZ was shown to induce Bcl-xL expression thereby protecting cardiomyocytes for doxorubicin cytotoxicity (Aguilar *et al*. 2014). In contrast to hematological malignancies, where GCs may reduce Bcl-xL expression thereby favoring apoptosis (Laane *et al*. 2007), in PCa it was shown that GCs upregulate Bcl-xL resulting in resistance to apoptosis-inducers (Petrella *et al*. 2006). We confirmed the upregulation of Bcl-xL in our docetaxel-resistant PCa cells, in line with a previous study (O'Neill *et al*. 2011), and show here that GR antagonism leads to a reduction in Bcl-xL expression, providing a potential mechanistic explanation for the restored sensitivity to docetaxel in resistant PCa cells.

Yet, we cannot fully attribute the resensitizing capacity of the GR antagonists to the GR, as both steroidal drugs (RU-486 and CPA) were reported to directly inhibit P-gp function (Gruol *et al*. 1994, Frohlich *et al*. 2004). In our studies, we compared RU-486 and CPA head-to-head to known P-gp inhibitor verapamil. Interestingly, at dosages with equal P-gp inhibition, both RU-486 and CPA outperformed verapamil, suggesting that mechanisms other than P-gp inhibition are responsible, i.e., the GR pathway. It is unclear, however, if an inhibitory effect on P-gp function underlies the sensitizing effects of RU-486 on paclitaxel and cisplatin reported in literature (Jurado *et al*. 2009, Skor *et al*. 2013). P-gp was shown to be expressed in the cell lines used (MDA-MB-231; Zhang *et al*. 2014) and HeLa (Al-Abd *et al*. 2011) respectively) but the issue is not discussed in these reports. Besides P-gp, GR antagonists were also described to affect progesterone receptor (PR) activity (Honer *et al*. 2003), although the effects of the GR antagonists on docetaxel sensitivity seem to be PR-independent since we observe sensitizing effects in both PR-positive (DU145 and 22Rv1; Lau *et al*. 2000, Hartel *et al*. 2004) and PR-negative cell lines (PC3; Lau *et al*. 2000).

DEX is routinely used in the treatment of patients with advanced PCa, although it actually may contribute to resistance to therapy. Indeed, the functional involvement of the GR in resistance to anti-AR therapy (i.e. enzalutamide) was demonstrated (Arora *et al*. 2013, Isikbay *et al*. 2014). Enhanced GR expression was observed in enzalutamide-resistant tumors *in vivo* and in tumor biopsies from enzalutamide-pretreated PCa patients (Arora *et al*. 2013). AR was shown to directly repress GR expression in PCa via a negative AR response element in the GR-promoter (Xie *et al*. 2015). It was proposed that GR is able to take over AR function due to a significant overlap in transcriptome. As a consequence, stimulation of the GR activity can rescue cells from enzalutamide-induced cell death (Arora *et al*. 2013).

We now demonstrate that the GR is upregulated in PCa tumors of patients treated with docetaxel. As GCs are frequently administered in combination with docetaxel, GC usage may counteract the antitumor efficacy of this chemotherapeutic agent. Alternatively, GC administration prior to treatment with chemotherapeutics agents may actually increase sensitivity to chemotherapeutics due to homologous downregulation of the GR (Rosewicz *et al*. 1988). Hence, phased timing (i.e. precise treatment sequencing) of GCs in combination with other treatment options (chemotherapy or anti-AR therapy) should be carefully considered. Reassuringly, a recent meta-analysis on the usage of GC prednisone showed no difference in overall survival in prednisone vs nonprednisone treated patients (Morgan *et al*. 2014).

Based on our preclinical results, combinational treatment with docetaxel and GR antagonists may be a promising therapeutic approach in docetaxel-resistant disease. Currently, CRPC patients that progress on docetaxel are routinely treated with AR- or AR-axis targeting drugs (i.e. abiraterone acetate or enzalutamide; de Bono *et al*. 2011, Scher *et al*. 2012). Not all patients, however, respond to therapeutic targeting of the AR-axis, in particular those with AR-V7-expressing tumors (Antonarakis *et al*. 2014). Although the clinical importance of AR-V7 in resistance to docetaxel is not confirmed (Antonarakis *et al*. 2015, Onstenk *et al*. 2015), preclinical studies do suggest that expression of AR-V7 promotes resistance to taxane-based chemotherapeutics (Thadani-Mulero *et al*. 2014, Zhang *et al*. 2015). We now show that combined GR antagonism and docetaxel treatment may also be effective in AR-V7-expressing, docetaxel-resistant PCa cells. Our study also reveals enhanced GR levels as a result of docetaxel treatment, and augmented GR may significantly undermine the efficacy of AR-targeting agents in patients that progress on docetaxel treatment (Arora *et al*. 2013). Based on this, GR antagonism may also be useful in combination with AR-targeting drugs. A phase I/II clinical study assessing combined treatment with enzalutamide and RU-486 is currently ongoing (NCT02012296) to identify the recommended dose of RU-486 and to monitor adverse effects and antitumor activity of this drug combination. It is interesting to note that RU-486 (as a mono treatment) was already assessed in a phase II clinical trial in CRPC patients in which a treatment schedule of 200 mg/day orally was very well tolerated (Taplin *et al*. 2008). In addition, other parameters influenced by GR activity, e.g., bone mineral density, were not influenced upon RU-486 treatment (Kettel *et al*. 1996). Taken together, the use of the GR inhibitors in CRPC patients is well tolerated and preclinical studies endorse further development towards clinical translation.

In summary, we believe that the clinical use of the GR antagonists could be beneficial in advanced PCa patients, as GR is often overexpressed. Combination treatment of the GR antagonists with docetaxel is worthwhile pursuing in order to enhance the antitumor efficacy of docetaxel in the treatment of patients with chemotherapy resistant PCa.

## Supplementary data

This is linked to the online version of the paper at http://dx.doi.org/10.1530/ERC-15-0343.

## Author contribution statement

J Kroon designed, carried out and analyzed the *in vitro* experiments and wrote the manuscript. M Puhr performed and analyzed the immunohistochemical study with the TMA and established the PC3-DR and DU145-DR cell lines. J T Buijs, G van der Horst, and D M Hemmer contributed to the data acquisition and interpretation. K A Marijt designed and cloned the CRISPR/CAS9 plasmids. M S Hwang, M Masood, and S Grimm carried out the western blot analysis of antiapoptotic proteins. J M Metselaar, G Storm, O C Meijer, and Z Culig provided invaluable intellectual input on the study design and concepts. G van der Pluijm supervised J Kroon, provided intellectual input and helped writing the manuscript. All co-authors improved the manuscript and approved its final version.
